# Initiation of Antiresorptive Drug Treatment during Endocrine Therapy for Breast Cancer—A Retrospective Cohort Study of 161,492 Patients in Germany

**DOI:** 10.3390/cancers15061847

**Published:** 2023-03-19

**Authors:** Niklas Gremke, Sebastian Griewing, Arturas Kadys, Karel Kostev, Uwe Wagner, Matthias Kalder

**Affiliations:** 1 Department of Gynecology and Obstetrics, University Hospital Marburg, Philipps-University Marburg, Baldingerstraße, 35043 Marburg, Germany; 2 Epidemiology, IQVIA, Main Airport Center, Unterschweinstiege 2–14, 60549 Frankfurt, Germany

**Keywords:** breast cancer, endocrine therapy, tamoxifen, aromatase inhibitors, antiresorptive therapy, bisphosphonates, denosumab, Germany

## Abstract

**Simple Summary:**

Endocrine therapy (ET), which significantly reduces breast cancer (BC) recurrence and mortality rates, is the primary systemic therapy for hormone receptor positive BC. However, in addition to frequently reported TAM- and AI-related side effects, such as hot flashes, arthralgias, and myalgias, treatment with AIs in particular leads to accelerated bone loss (AI-associated bone loss, AIBL) and increased bone fracture risk. To the best of our knowledge, little is known about the prescription spectrum of antiresorptive drugs in BC patients treated with ET in Germany. To explore this further, we conducted a retrospective cohort study that included 161,492 patients under ET to measure the cumulative incidence of antiresorptive drug prescription for TAM and AIs and estimate the relationship between initial drug (AIs versus TAM) and antiresorptive drug prescription. Finally, our study provides an overview of the most frequently prescribed antiresorptive drugs in Germany.

**Abstract:**

Background: The aim of this retrospective cohort study was to measure the proportion of women with an initial prescription of an antiresorptive drug (bisphosphonates or denosumab) during five years of endocrine breast cancer therapy. Methods: The study included women with an initial prescription of tamoxifen (TAM) or aromatase inhibitors (AIs) between January 2016 and December 2020. Kaplan–Meier analyses were performed to show the cumulative incidence of antiresorptive drug prescription for TAM and AIs separately for four age groups. A univariable Cox proportional hazards regression model was also used to estimate the relationship between initial endocrine drug (AIs vs. TAM) and antiresorptive drug prescription. Results: Within 5 years, 14.1% of patients on AI and 6.1% on TAM received their first prescription for an antiresorptive drug (*p* < 0.001). The difference between AI and TAM was greatest in women ≤50 years (12.9% of AI and 2.8% of patients on TAM), and smallest in women >80 years (14.5% of AI and 10.3% of patients on TAM). The proportion of denosumab was 46.2% among AI patients vs. 29.1% among patients on TAM (*p* < 0.001) as alendronate was prescribed to 36.9% of AI vs. 50.0% of patients on TAM. Conclusions: Across all age groups, the cumulative incidence of antiresorptive drug prescriptions was higher in patients with BC treated with AI than those receiving TAM. Denosumab was most frequently used as an antiresorptive drug in patients treated with AI, while alendronate was administered more often in patients treated with TAM.

## 1. Introduction

Breast cancer, which affects around two million women globally every year, is the most prevalent type of cancer. In Germany alone, it is estimated to cause approximately 72,000 new cases annually [1]. Based on molecular features (e.g., expression of estrogen receptor (ER), progesterone receptor (PR), human epidermal growth factor receptor 2 (HER2), and the proliferation marker Ki-67) BC is divided into different molecular subtypes, with the hormone receptor positive (ER and PR+) being by far the most common subtype (60–80% of all BC cases) [2]. Endocrine therapy (ET), which counteracts estrogen-promoted tumor growth and therefore significantly reduces BC recurrence and mortality rates [3,4,5], is the primary systemic therapy for HR+ positive BC. ET is routinely administered for 5 years, whereby the type of antiestrogen therapy depends on menopausal status, tolerability, and the patient’s risk of BC recurrence [6]. As a selective estrogen receptor modulator (SERM) with antagonistic properties in the breast and paradoxically agonistic functions in the uterine endometrium, bone, and cardiovascular system, tamoxifen is administered in both pre- and postmenopausal BC patients [3,7]. However, aromatase inhibitors (steroidal: exemestane, non-steroidal: anastrozole and letrozole) have largely replaced TAM in the treatment of postmenopausal BC patients due to better efficacy and fewer toxicities, such as the induction of uterine cancer or thromboembolic events [8,9]. More specifically, AIs block the conversion of androgens to estrogens in peripheral tissues, local malignant and normal breast tissue, decrease plasma estrogen levels and are only effective in postmenopausal women (including premenopausal women who are postmenopausal due to ovarian suppression with GnRH analogues or ovariectomy) [4,10,11]. From a clinical perspective, postmenopausal BC patients, especially those with lobular breast cancers or a high risk of BC recurrence, should receive AI as an initial treatment. Postmenopausal BC patients with high age, very low risk of recurrence, or those with contraindications for AIs, may be considered for TAM treatment [12].

However, all of these proven survival efficacies of TAM and AIs come at a cost. In addition to frequently reported TAM- and AI-related side effects, such as hot flashes, arthralgias, and myalgias, treatment with AIs in particular leads to accelerated bone loss (AI-associated bone loss, AIBL) and increased bone fracture risk [13,14]. Mechanistically, complete AI-mediated estrogen depletion disturbs the dynamic balance of bone resorption and new bone formation within the bone remodeling compartment, and accelerates bone resorption leading to a two- to four-fold increase in bone loss compared to physiological postmenopausal low bone mineral density (BMD) loss [15,16,17]. Notably, the fracture incidence of BC patients treated with AI is approximately 18–20% after 5 years of follow up, and BC patients hospitalized with a bone fracture showed a higher risk of death (HR = 1.83; 95% CI: 1.50–2.22) than those without bone fracture, leading to increased morbidity and mortality [18,19,20]. In view of this issue, there was an urgent need to establish a risk factor assessment and a treatment algorithm for AIBL in women with BC. Using this recommended treatment algorithm, a specific antiresorptive therapy is indicated when the T-score is less than 2, or when two of the following risk factors are present: age > 65 years, T-score < 1.5, smoking, body mass index < 20, family history of hip fracture, personal history of fragility, fracture, and age > 50 years or oral glucocorticoid use for >6 months [21]. If an antiresorptive treatment is recommended, bisphosphonates (alendronate, risedronate, ibandronate, or zoledronate) or denosumab are the suggested first line treatment on AIBL [22]. Particularly, upon binding to bone mineral, the bisphosphonates are internalized into osteoclasts by endocytosis. Nitrogen-containing bisphosphonates (all bisphosphonates mentioned above) inhibit the farnesyl pyrophosphate synthase (FPPS), thereby preventing the prenylation of small GTPase proteins essential for the function and survival of osteoclasts [23,24]. Denosumab is a monoclonal antibody against the receptor activator of nuclear factor κB ligand (RANKL), an essential mediator of osteoclast formation, function, and survival. More in detail, denosumab binds with high affinity and specificity to RANKL, thereby inhibiting osteoclast-mediated bone resorption, as well as osteoclast maturation and survival. Several in vivo studies reveal that inhibition of RANKL leads to improved bone geometry and increased bone density and strength [25]. However, in addition to specific pharmacological treatments, lifestyle changes that promote bone health (e.g., increasing physical activity) and further measures, such as vitamin D supplementation and sufficient calcium intake, are also recommended for patients receiving ET.

To the best of our knowledge, little is known about the prescription of different antiresorptive drugs in BC patients treated with ET in Germany. Aiming to explore this topic more thoroughly, we conducted a retrospective cohort study that included 161,492 patients under ET to measure the cumulative incidence of antiresorptive drug prescription for TAM and AIs and estimate the relationship between initial drug (AIs versus TAM) and antiresorptive drug prescription.

## 2. Materials and Methods

### 2.1. Database

The retrospective cohort study being described utilized the IQVIA longitudinal prescription database (LRx) [26]. This database encompasses around 80% of prescriptions that are reimbursed by statutory health insurance funds in Germany. Patient-level data, including patient age and gender, are available. To ensure data privacy in compliance with regulations, all patient information has been fully anonymized by the data provider. The database includes full details of each prescription, such as the product brand, substance, package size, and product form, as well as dispensing dates. However, it does not include information on diagnoses or laboratory tests [26]. Previous studies on pharmacoepidemiology have also utilized this database [27,28].

### 2.2. Study Population and Outcomes

This study retrospectively analyzed women who were initially prescribed tamoxifen or aromatase inhibitors (anastrozole, letrozole, exemestane) between January 2016 and December 2020 (index date). The study aimed to measure the proportion of women who received an initial prescription of an antiresorptive drug (such as bisphosphonates or denosumab) during endocrine breast cancer therapy, within a timeframe of up to five years from the index date. Women who had been prescribed antiresorptive drugs prior to or on the index date were excluded from the study. Each patient was followed for up to 60 months from the index date until they received their first prescription of an antiresorptive drug or until their therapy with tamoxifen and aromatase inhibitors ended, switched, or discontinued. Discontinuation of therapy was defined as a period of at least 180 days without therapy. The duration of each prescription was calculated based on the package size, number of packages, and defined daily dose (DDD).

### 2.3. Statistical Analyses

Women with tamoxifen therapy were matched (1:1) to those with aromatase inhibitors using a propensity score based on age on the index date, index year, and prescriptions of calcium and vitamin D within 12 months prior to the index date. Further analyses were conducted for matched pairs.

Kaplan–Meier analyses were performed to show the cumulative incidence of antiresorptive drug prescription for tamoxifen and aromatase inhibitors, separately, for four age groups (≤50, 51–60, 61–70, 71–80, and >80 years). A univariable Cox proportional hazards regression model was also used to estimate the relationship between initial drug (aromatase inhibitors versus tamoxifen) and antiresorptive drug prescription. *p*-values < 0.01 were considered statistically significant. Analyses were carried out using SAS version 9.4 (SAS institute, Cary, NC, USA).

## 3. Results

### 3.1. Basic Characteristics of the Study Sample

This study included 161,492 patients. Of these, 80,746 received aromatase inhibitors and 80,746 tamoxifen as an initial endocrine therapy. The baseline characteristics of the study population are shown in Table 1. The mean (standard deviation) age was 64.4 (SD: 12.5) years and the majority of patients were treated by gynecologists (77.4% patients on TAM and 71.7% on AI), with oncologists treating 5.6% of patients on TAM and 10.7% of those on AI. Prior to the index date, 6.8% of study patients received prescriptions for calcium or vitamin D.

### 3.2. Incidence of Antiresorptive Therapy

**Figure 1:**
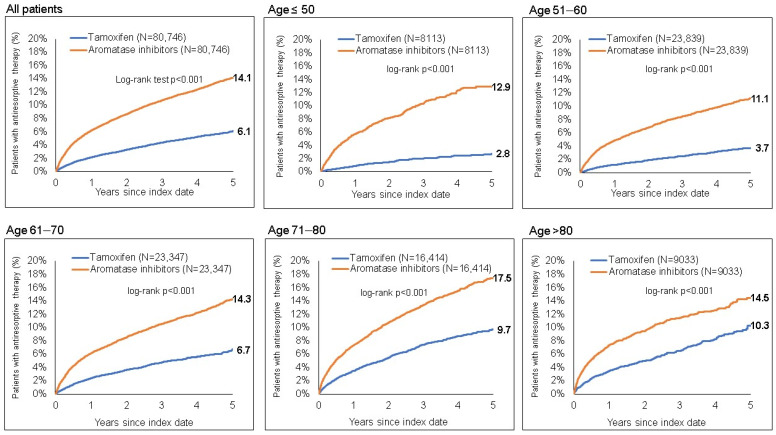
Cumulative incidence of antiresorptive drug prescription among women treated with TAM and AIs.

Just 14.1% of AI and 6.1% of patients on TAM received their first antiresorptive drug prescription (*p* < 0.001) (Figure 1) within 5 years after the index date. The difference between AI and TAM was greatest in women ≤50 years (12.9% on AI and 2.8% of patients on TAM), and smallest in women >80 years (14.5% of patients on AI and 10.3% on TAM). The results of the Cox regression models are shown in Table 2. AI was associated with a higher risk of antiresorptive therapy prescription than TAM (HR: 2.54, *p* < 0.001). Interestingly, the degree of association decreased with age from HR: 5.55 (*p* < 0.001) among women aged ≤50 years to HR: 1.86 (*p* < 0.001) among women >80 years (Table 2).

### 3.3. Antiresorptive Drugs Prescribed

**Figure 2:**
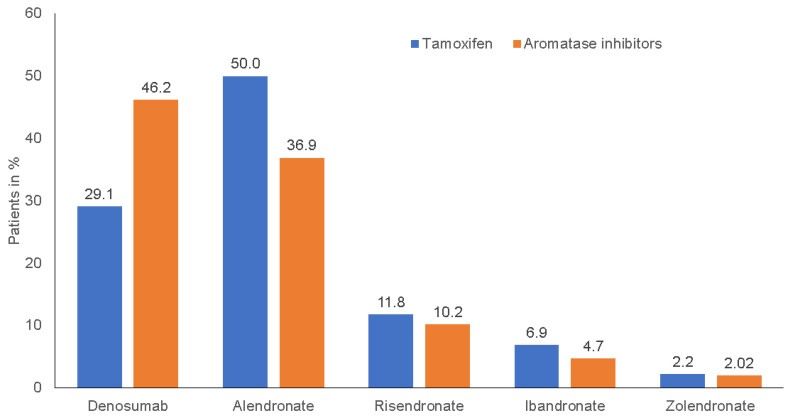
Antiresorptive drugs prescribed in women treated with TAM and AIs (100% = patients with at least one antiresorptive drug prescription).

Figure 2 shows the proportions of antiresorptive drugs prescribed among women receiving TAM and AI therapy. The proportion of denosumab was 46.2% among patients on AI vs. 29.1% on TAM (*p* < 0.001) as alendronate was prescribed among 36.9% of patients on AI vs. 50.0% on TAM. The differences for other antiresorptive drugs were smaller and not significant.

## 4. Discussion

Our retrospective cohort study of 161,492 BC patients in Germany showed that 14.1% of patients on AI and 6.1% of patients on TAM received their first antiresorptive drug prescription within 5 years of ET initiation (*p* < 0.001). In line with these figures, Cox regression models revealed that AI treatment was associated with a higher risk of antiresorptive therapy prescription than TAM treatment (HR: 2.54, *p* < 0.001). These observed differences can be interpreted in the context of the current literature. It is a well-established fact that physiological postmenopausal bone loss is approximately 1–2% per year, whereas AI treatment leads to increased bone loss of 2–3% per year in postmenopausal women [29,30]. Furthermore, AIBL is associated with an increased risk of fractures, as well as reducing patient quality of life and leading to increased morbidity and mortality [13,31]. In the past, several studies have analyzed the effects of AIs and TAM on bone health in patients with hormone receptor-positive BC. In this context, the ATAC (Arimidex, tamoxifen, alone or in combination) trial revealed that AI treatment for five years led to bone loss of 6.1% at the lumbar spine and 7.2% at the hip, whereas TAM has a protective effect on bones with bone mineral density (BMD) increasing by 2.8% and 0.7%, respectively, in patients receiving the treatment [30,32]. In addition, the BIG1-98 study showed a higher fracture rate among postmenopausal BC patients receiving letrozole compared to those treated with TAM (9.3% vs. 6.5%, *p* < 0.001, RR: 1.38, 95% CI: 1.13–1.69), with the wrist being the most common site of fracture [16]. In summary, these data support our findings of a higher antiresorptive drug incidence in BC patients treated with an AI compared to those with TAM treatment (Figure 1).

Interestingly, we also observed that the difference between AI and TAM in terms of antiresorptive drug prescription was greatest in women ≤50 years (12.9% of AI and 2.8% of TAM patients) and smallest in women >80 years (14.5% of AI and 10.3% of TAM patients). The increased cumulative incidence of antiresorptive drug prescription among women treated with TAM aged >80 years seems surprising, since it is known that TAM exerts agonistic effects on the bone exclusively in postmenopausal women, whereas the agonist effects of TAM are insufficient to prevent bone loss in healthy premenopausal women [33]. One of the limitations of our study was that no information regarding co-diagnoses was available. Therefore, it is conceivable that the osteo-neutral effect of TAM in elderly patients is undermined by various mechanisms of age-related bone loss, such as osteoporosis that has manifested prior to the primary diagnosis of BC, increased parathormone (PTH) levels due to impaired renal function, decline in physical activity, and secondary hyperparathyroidism. In particular, both calcium and vitamin D deficiency can contribute to secondary hyperparathyroidism [34]. Despite including prescriptions of calcium and vitamin D within the propensity score of this retrospective cohort study, it is unclear whether older patients take these medications reliably.

Besides this, antiresorptive therapy is not indicated for all BC patients receiving ET with AIs. A lot of scientific societies have published clinical guidelines for managing bone health in women with BC receiving aromatase inhibitor therapy. To summarize briefly, there are three potential indications for initiating antiresorptive treatment: to reduce ET-induced bone loss, to reduce the risk of developing bone metastases, and to reduce the occurrence of bone metastases [32]. Given this variety of indications, we wish to address the fact that our study outcome measure (proportion of women with an initial prescription of antiresorptive drugs during the ET within TAM or AI) is not equivalent to the outcome for newly diagnosed AIBL, bone fractures, osteoporosis, or bone metastases. If antiresorptive therapy is indicated, several treatment options are currently available to maintain bone health in patients undergoing AI therapy. The two main bone-targeted therapies to counteract bone loss are oral (alendronate and risedronate) and i.v. (zoledronate and ibandronate) bisphosphonates, as well as monoclonal antibodies (denosumab). Based on current evidence, recently published papers recommend subcutaneous denosumab (60 mg twice yearly) and intravenous zoledronate (4 mg q6mo) as the preferred agents for the prevention and treatment of AIBL [35,36,37,38]. Zoledronate should be preferred in cases where effects on disease recurrence are the priority, and denosumab when fracture risk is the dominant concern [13]. In our study, we found that denosumab was prescribed most frequently, being administered in 46.2% of AI-treated patients, whereas zoledronate was only used in 2% of AI-treated patients (Figure 2). With regard to costs, while zoledronate is indeed less expensive than denosumab, the therapy is not suitable for all patients (e.g., those with renal insufficiency), which may explain this difference. In particular, we can also show that the majority of BC patients under ET were treated by gynecologists, followed by oncologists and general practitioners (Table 1). In fact, in Germany, BC patients are generally treated by gynecologists rather than oncologists and general practitioners, with gynecologists receiving special training and regular updates regarding BC treatment [27]. In view of the complex issue of ET and bone health, a multidisciplinary management approach together with bone specialists may be necessary to implement the latest guideline recommendations in clinical practice. Nevertheless, for patients with BC on TAM-treatment, the most frequently prescribed antiresorptive therapy in Germany was alendronate (50% of patients on TAM), which has been shown to have positive effects on BMD in patients receiving ET and is recommended in recently published treatment guidelines [39].

The present study has several limitations that need to be acknowledged. The primary constraint is the lack of some important variables in the German longitudinal prescription database (LRx database), which is not tailored to specific research inquiries. A thorough examination is necessary to determine if the available data can yield a valid response to the research question. In particular, the prescription database utilized in the study lacks information concerning diagnoses and TNM status. As a result, it was not feasible to stratify the data based on cancer stages or examine disease severity or co-diagnoses. Additionally, there is no information available on prior breast cancer treatments, such as chemotherapy or procedures such as ovariectomy and GnRH agonists, which could also impact bone metabolism. Secondly, the absence of mortality data to evaluate the reasons for loss of follow-up is another limitation. Loss of follow-up could be due to various reasons such as death, change of insurance provider, or relocation. Thirdly, there is a lack of in-patient and phenotypic data, such as therapeutic outcomes, comorbidities, and adverse drug reactions. Fourthly, the prescription database used for this study does not include important lab values, preventing its use in drug safety analyses. Fifthly, the analyses conducted using the LRx database are retrospective and no conclusions can be drawn about potential confounding variables, such as population bias, disease severity, prevalent complications, or other individual circumstances [26].

Despite these limitations, the study has several strengths, such as a vast number of patients, an extended observation period, and nationally representative data on drug prescription.

## 5. Conclusions

In summary, our large retrospective cohort study provides a good overview of the most frequently prescribed antiresorptive drugs in Germany and their incidence under ET in patients with BC receiving TAM or AI treatment in Germany.

## Figures and Tables

**Table 1:** Basic characteristics of the study sample.

Variable	Proportion among Patients Treated with TAM (%)	Proportion among Patients Treated with AIs (%)	*p*-Value
N	80,746	80,746	
Age (Mean, SD)	64.4 (12.5)	64.4 (12.5)	1.000
Age ≤ 50	8113 (10.1)	8113 (10.1)	1.000
Age 51–60	23,839 (29.5)	23,839 (29.5)	
Age 61–70	23,347 (28.9)	23,347 (28.9)	
Age 71–80	16,414 (20.3)	16,414 (20.3)	
Age > 80	9033 (11.2)	9033 (11.2)	
Prescription for calcium/vitamin D within 12 months prior to index date	5464 (6.8)	5464 (6.8)	1.000
Physician initiating therapy			
Gynecology	62,520 (77.4)	57,862 (71.7)	<0.001
Oncology	4499 (5.6)	8.640 (10.7)	
General practitioner	5737 (7.1)	4437 (5.5)	
Others or unknown	7990 (9.9)	9807 (12.1)	

Proportions of patients are given in % unless otherwise indicated. SD: standard deviation.

**Table 2:** Association between AIs and antiresorptive therapy initiation compared to TAM (Cox regression models).

Cohort	HR (95% CI) for AI Compared to TAM	*p*-Value
Total	2.54 (2.42–2.66)	<0.001
Age ≤ 50	5.55 (4.53–6.79)	<0.001
Age 51–60	3.56 (3.21–3.96)	<0.001
Age 61–70	2.39 (2.20–2.59)	<0.001
Age 71–80	1.98 (1.82–2.15)	<0.001
Age > 80	1.86 (1.65–2.11)	<0.001

## Data Availability

Anonymized raw data are available upon reasonable request.
